# 

**Published:** 1818-01

**Authors:** 


### LONDON MEDICAL AND PHYSICAL JOURNAL;

CONTAINING

ORIGINAL CORRESPONDENCE of EMINENT PRACTITIONERS,

AND THE

EARLIEST INFORMATION ON SUBJECTS CONNECTED WITH

MEDICINE, SURGERY, CHEMISTRY, PHARMACY, BOTANY, AND NATURAL HISTORY.

---

*Successively conducted by*

T. BRADLEY, M.D.

A. F. M. WILLICH, M.D.

R. BATTY, M.D.

A. A. NOEHDEN, M.D.

J. ARNEMAN, M.D.

J. P. PFAFF, M.D.

J. ADAMS, M.D.

W. SHEARMAN, M.D.

W. ROYSTON, Esq.

AND

SAMUEL FOTHERGILL, M.D.

Assisted by eminent Gentlemen, in the various Branches of the Profession.

---

5145

VOL. XXXIX.

FROM JANUARY TO JUNE, 1818.

---

Et quoniam variant morbi, variabimus artes;
Mille mali species, mille salutis erunt.

---

*LONDON:*

PRINTED FOR THE PROPRIETORS, BY J. ADLARD, 23, BARTHOLOMEW-CLOSE, AND 39, DUKE-STREET, SMITHFIELD.

PUBLISHED BY J. SOUTER, NO. 73, ST. PAUL'S CHURCH-YARD; AND MAY BE HAD OF ALL BOOKSELLERS.

W1
LO 31

### CATALOGUE OF MEDICAL BOOKS.

A Critical Inquiry into the Nature and Treatment of the Case of Her Royal Highness the Princess Charlotte of Wales, and her Infant Son; with the probable Causes of their Deaths, and the subsequent Appearances. By Rees Price, Member of the Royal College of Surgeons, London. 8vo. 3s. 6d.

On Diagnosis. Part I. By Marshall Hall, M.D. 8vo. bds. 4s.

A Second Letter on the Necessity of a Public Inquiry into the Cause of the Death of Her Royal Highness the Princess Charlotte and her Infant; by Jesse Foot, Esq. 8vo. 6d.

Medico-Chirurgical Transactions. Vol. VIII. Part II. 8vo. boards, 10s. 6d.

An Essay on the Disorders of Old Age, and on the Means for prolonging Human Life; by A. Carlisle, F.R.S. &c. 8vo. bds. 5s.

Transactions of the Association of Fellows and Licentiates of the King's and Queen's College of Physicians in Ireland. 8vo, boards, 14s.

Observations on the Treatment of certain severe Forms of Hæmorrhoidal Excrescence; by J. Kirby, A.B. Member and one of the Censors of the Royal College of Surgeons in Ireland, &c. 8vo. sewed, 3s.

The Code of Health and Longevity; or, a General View of the Rules and Principles calculated for the Preservation of Health and the Attainment of long Life. By the Right Hon. Sir John Sinclair, Bart. 8vo. plates, boards, 1l. 1s.

The Chemical Catechism; with Notes, Illustrations, and Experiments. By Samuel Parkes, F.L.S. &c. &c. The eighth Edition, greatly enlarged. 8vo. boards. 14s.

The Influence of the Atmosphere, more especially the Atmosphere of the British Isles, on the Health and Functions of the Human Frame; by James Johnson, M.D. 8vo. boards, 10s.

A Disquisition on the Stone and Gravel; by S. Perry, surgeon.

An Account of some Experiments made with the Vapour of Boiling Tar, in the Cure of Pulmonary Consumption; by Alex. Crichton, M.D. F.R.S. 2s. 6d.

---

### *NOTICES TO CORRESPONDENTS.*

*The Proprietors have received information from the Stamp-office, that, in future, Notices of Lectures, with the* Names *or* Address *of the Lecturers annexed, will be charged with the Advertisement-duty. They are, therefore, under the necessity of omitting names and address, unless 5s. is paid with each notice. They regret, in common with their subscribers, this additional tax, however light, on a profession already but ill requited for their services to the public.*

*Our* Constant Reader *is never tired of his favourite subject. But, by this time he must understand that we admit no anonymous severities against Gentlemen who favour us with their names. We are ready to admit our lack of wisdom, but think C. R. an incompetent judge of candour.*

*Our correspondent at Northamptonshire may be assured, that the only reason we have not answered his enquiry is, that we wait for* authentic *information, which is promised us.*

